# Bile duct reconstruction following laparoscopic cholecystectomy in England

**DOI:** 10.1007/s00464-015-4641-8

**Published:** 2016-01-29

**Authors:** Y. El-Dhuwaib, J. Slavin, D. J. Corless, I. Begaj, D. Durkin, M. Deakin

**Affiliations:** The Institute for Science and Technology in Medicine, Keele University, Stoke-on-Trent, UK; Department of Surgery, Royal Stoke University Hospital, Stoke-on-Trent, ST4 6RG UK; Health Informatics Department, University Hospitals Birmingham, Birmingham, UK; Department of Surgery, Mid Cheshire Hospitals NHS Foundation Trust, Crewe, UK

**Keywords:** Bile duct injury, Bile duct reconstruction, Hospital Episode Statistics data, Laparoscopic cholecystectomy, On-table cholangiography

## Abstract

**Objectives:**

To determine the incidence of bile duct reconstruction (BDR) following laparoscopic cholecystectomy (LC) and to identify associated risk factors.

**Background:**

Major bile duct injury (BDI) requiring reconstruction is a serious complication of cholecystectomy.

**Methods:**

All LC and attempted LC operations in England between April 2001 and March 2013 were identified. Patients with malignancy, a stone in bile duct or those who underwent bile duct exploration were excluded. This cohort of patients was followed for 1 year to identify those who underwent BDR as a surrogate marker for major BDI. Logistic regression was used to identify factors associated with the need for reconstruction.

**Results:**

In total, 572,223 LC and attempted LC were performed in England between April 2001 and March 2013. Five hundred (0.09 %) of these patients underwent BDR. The risk of BDR is lower in patient that do not have acute cholecystitis [odds ratio (OR) 0.48 (95 % CI 0.30–0.76)]. The regular use of on-table cholangiography (OTC) [OR 0.69 (0.54–0.88)] and high consultant caseload >80 LC/year [OR 0.56 (0.39–0.54)] reduced the risk of BDR. Patients who underwent BDR were 10 times more likely to die within a year than those who did not require further surgery (6 vs. 0.6 %).

**Conclusions:**

The rate of BDR following laparoscopic cholecystectomy in England is low (0.09 %). The study suggests that OTC should be used more widely and provides further evidence in support of the provision of LC services by specialised teams with an adequate caseload (>80).

Laparoscopic cholecystectomy (LC) is a common operation, with over 60,000 operations undertaken each year in England. Based on conversion rate, it has been suggested that LC should be undertaken by high-volume surgeons [1].

Bile duct injury (BDI) is a rare but serious complication of cholecystectomy, and the reported incidence following LC is between 0.1 and 1.5 % [2–8]. Gallrick et al showed that the overall incidence of BDI was 1.5 %; however, they included patients with bile leaks, partial duct injury, and non-specific injuries that would not have required reconstruction. The rate decreases to 0.1 % if only the most serious cases of BDI are included [6]. BDI is associated with significant morbidity and mortality. Early complications include collections or peritonitis and if not treated sepsis, multiorgan failure and death [9]. Patients who sustain a BDI are also at risk of long-term problems including strictures, cholangitis and secondary biliary cirrhosis, requiring multiple hospital admissions, a shortened life expectancy and transplantation. The reported perioperative mortality rate following BDI varies between 0 and 7.2 % [5, 10–12] with a 1-year mortality of 3.9 % [6]. A review of the literature showed (602 BDI from 15 studies) that the adjusted hazard ratio of death in the longer term in those sustaining BDI compared to those without BDI following LC or attempted LC was 2.79 (95 % CI 2.77–2.81) [4].

This study investigates bile duct reconstruction (BDR) following LC or attempted LC in England as a surrogate marker for major bile duct injury requiring reconstruction.

## Methods

Hospital Episode Statistics (HES) data were obtained from the National Health Service Information Centre (NHSIC) and imported into Microsoft SQL server for analysis. All patients who underwent LC or attempted LC between April 2001 and March 2013 were identified by searching the operative fields for the OPCS-4 (Office of Population Censuses and Surveys 4) codes J18* (cholecystectomy) and the corresponding laparoscopic codes.

Using diagnostic codes, International classification of Diseases version 10 (ICD 10), patients undergoing surgery for benign biliary disease of the gall bladder were identified. Those who underwent LC or attempted LC for a stone in the bile duct or for a malignant neoplasm of the liver, gall bladder, biliary tree or pancreas were excluded.

**Fig. 1:**
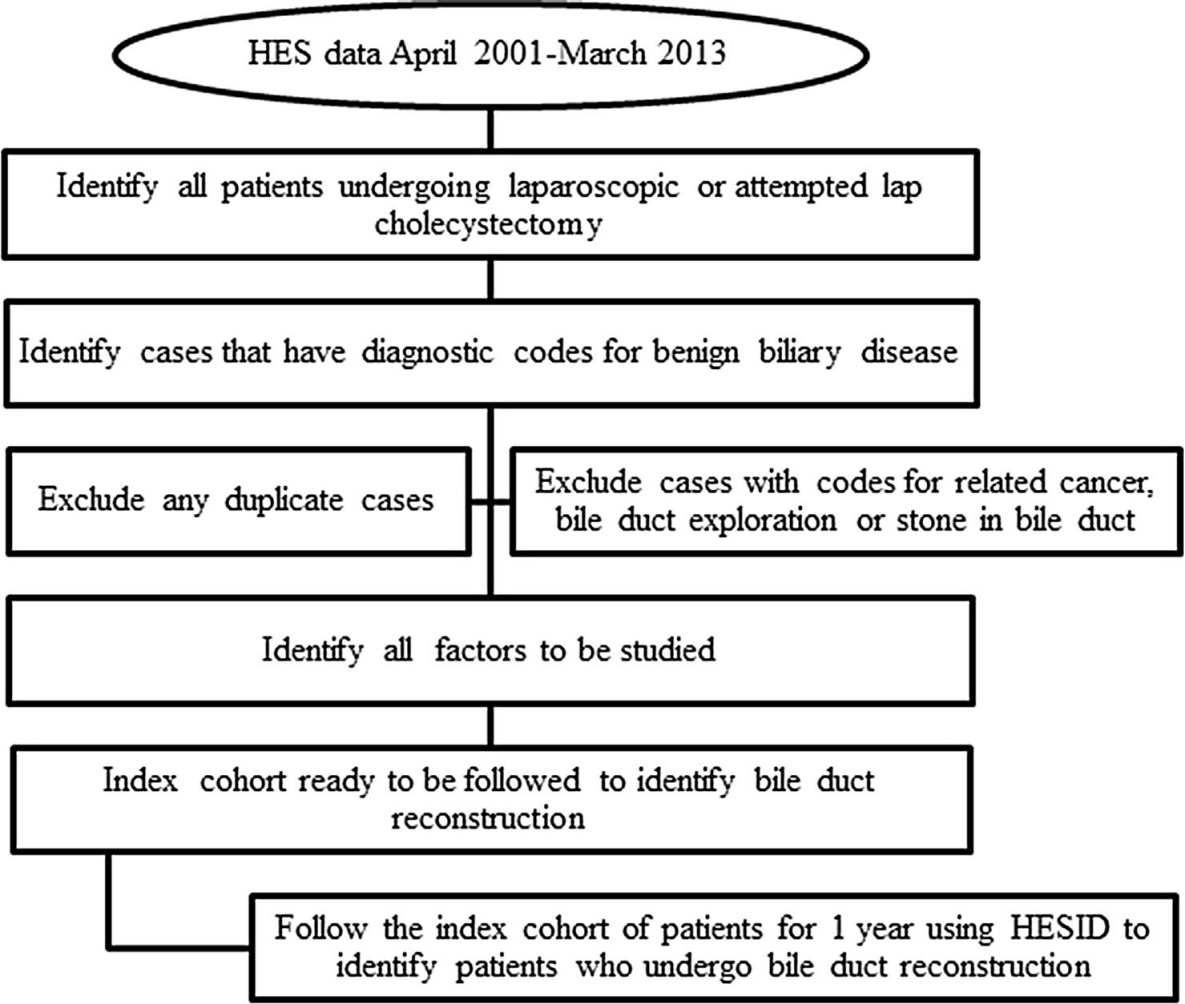
Study design

There is no specific code for BDI in either ICD-10 or OPCS-4; therefore, operative codes that are used for BDR were used to identify patients who required biliary reconstruction following LC or attempted LC. The cohort of patients was followed using HESID (a unique identifier for each patient in HES) to identify patients who underwent BDR within a year of the index operation. If a patient underwent more than one BDR, only the first operation was included in the analysis. A flow chart of the methods is shown in Fig. 1, and all codes used are summarised in Table 1.

**Table 1:** Operative and diagnostic codes used in this study

Codes used for cholecystectomy	
J181	Total cholecystectomy and surrounding tissue
J183	Total cholecystectomy
J185	Partial cholecystectomy
J188	Other excision of gall bladder
J189	Unspecified excision of gall bladder
Codes used for intraoperative cholangiography	
J372	Operative cholangiography through cystic duct
J373	Direct puncture operative cholangiography
Codes used for laparoscopic surgery and conversion	
Y718	Failed minimal access approach converted to open (before 2006)
Y714	Failed minimal access approach converted to open (after 2006)
Y508	Laparoscopic approach to abdominal cavity (before 2006)
Y75*	Laparoscopic approach to abdominal cavity (assisted, robotic, hand-assisted and other approach) (after 2006)
Codes used for diagnosis	
K800	Calculus of gall bladder with acute cholecystitis
K801	Calculus of gall bladder with other cholecystitis
K802	Calculus of gall bladder without cholecystitis
K808	Other cholelithiasis
K810	Acute cholecystitis
K811	Chronic cholecystitis
K818	Other cholecystitis
K819	Unspecified cholecystitis
K82*	Other diseases of gall bladder
K832	Perforation of bile duct
K85*	Acute pancreatitis
Codes used for exclusion in the diagnosis fields	
K803	Calculus of bile duct with cholangitis
K804	Calculus of bile duct with cholecystitis
K805	Calculus of bile duct without cholecystitis or cholangitis
K830	Cholangitis
K823	Fistula of gall bladder
K831	Obstruction of bile duct
K833	Fistula of bile duct
C22*	Malignant neoplasm of liver and intrahepatic duct
C23	Malignant neoplasm of gall bladder
C24*	Malignant neoplasm of other parts biliary tract
C25*	Malignant neoplasm of pancreas
Codes used for exclusions in the operative fields	
J182	Total cholecystectomy and exploration of common bile duct
J184	Partial cholecystectomy and exploration of common bile duct
Codes used to identify bile duct reconstruction	
J27.2	Partial excision of bile duct and anastomosis of bile duct to duodenum
J27.3	Partial excision of bile duct and anastomosis of bile duct to jejunum
J27.4	Partial excision of bile duct and end-to-end anastomosis of bile duct
J29.1	Anastomosis of hepatic duct to transposed jejunum and insertion of tubal prosthesis HFQ
J29.2	Anastomosis of hepatic duct to jejunum NEC
J30.1	Anastomosis of common bile duct to duodenum
J30.2	Anastomosis of common bile duct to transposed jejunum
J30.3	Anastomosis of common bile duct to jejunum NEC
J32.1	Reconstruction of bile duct
J32.2	Reanastomosis of bile duct

Factors that may affect the risk of BDR were divided into patient and non-patient groups. Patient-related factors studied included age, gender, acute pancreatitis, acute cholecystitis, comorbidity and deprivation index score. The Charlson comorbidity score was calculated using methods described by Dr Foster [13]. The deprivation index score was used as described in the English indices of deprivation [14].

Non-patient-related factors included were consultant caseload, hospital volume, consultant conversion rate, whether a trust was a regional hepatopancreatobiliary (HPB) centre and consultant rate of use of intraoperative cholangiography (IOC). Definitions are summarised in Table 2.

**Table 2:** Definitions used in this study

Factors	Definitions
Non-patients related factors	
Consultant caseload	Total number of operations performed under the care of a consultant in the previous year
Consultant conversion rate	Number of laparoscopic cholecystectomies converted divided by the total number of LC and attempted LC under the care of that consultant in the previous year
Hospital volume	Total number of laparoscopic cholecystectomies performed by an NHS Trust in the previous year
Consultant rate of on-table cholangiography (OTC)	Number of OTC’s performed by a consultant divided by the total number of LC attempted under the care of that consultant in the previous year
Patient-related factors	
Acute cholecystitis	Patients admitted as an emergency with diagnostic codes K800 or K810 who undergo cholecystectomy on that admission
Acute pancreatitis	Patients admitted as an emergency with a diagnostic code of K85* who undergo cholecystectomy on that admission
Major bile duct injury	Patient who underwent bile duct reconstruction within a year of index admission, i.e., hepaticojejunostomy, hepaticoduodenostomy, or resection of injured bile duct and reanastamosis

Mortality was assessed for all patients using data derived from the Office of National Statistics. One-year mortality was then calculated for patients with or without BDR.

### Statistics

Univariate analysis and multivariate analysis (logistic regression) were used to investigate which factors are associated with a risk of bile duct reconstruction.

**Fig. 2:**
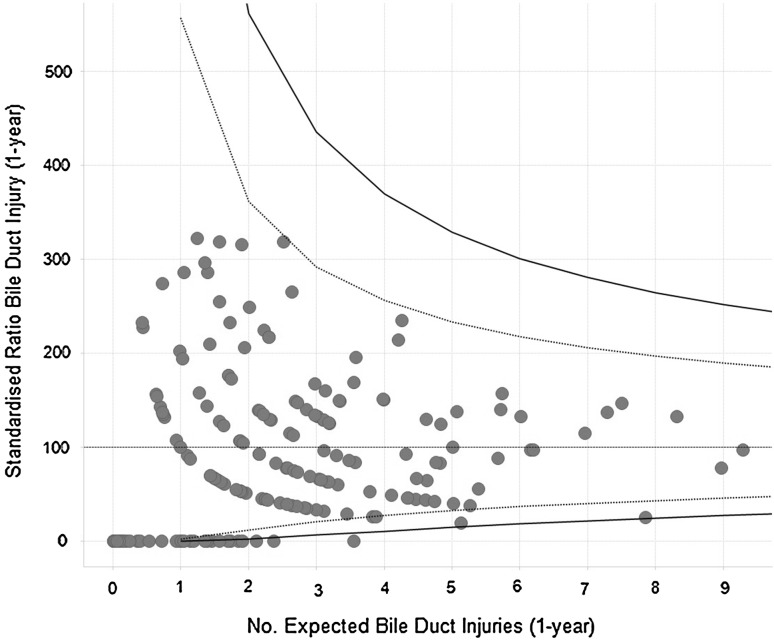
Funnel plot of Institutional Standardised Ratio for BDR following LC or attempted LC

A funnel plot was used to examine institutional variation and shows the standardised ratio of BDRs at 1 year following LC plotted against the number of expected BDRs (Fig. 2). The expected number of BDRs is derived using a multivariate logistic regression model that accounts for patient-related factors. The BDR ratio was calculated by dividing observed BDR per year over expected BDR per year multiplied by 100. Each hospital is represented by a blue dot. The dotted lines show the lower and upper 95 % control limit and the solid lines the upper and lower 99.8 % control limit as described by Eayers [15]. If a hospital falls outside the 99.8 % control limit, this is considered to be the result of special cause variation and would usually require further investigation.

## Results

 In total, 572,223 LCs and attempted LCs were performed in England between April 2001 and March 2013 (Table 3). More than half (56 %) were undertaken in patients under 55 years of age, while 7.2 % were performed in patients above 75 years. Just over three quarters of LCs or attempted LCs were undertaken in females. The majority of LCs was performed electively (89 %). Almost a third of emergency LCs were performed for acute cholecystitis and 13.3 % for acute pancreatitis. The number of LCs performed in the NHS in England almost doubled from 32,086 in 2001/2002 to 62,020 during 2012/2013. The overall conversion rate of LC in England is 4.3 %. One-year mortality rate following LC in England is 0.6 %. Around half of the patients who underwent LC or attempted LC had their surgery under the care of a consultant surgeon who performs between 20 and 80 cases a year, and a quarter of patients underwent surgery under care of consultants who perform less than 20 or more than 80 cases a year.

**Table 3:** Demographics of study cohort

	No. of cholecystectomies	Bile duct reconstruction within 1 year	%
Total	572,233	500	0.09
Age group			
<55	319,632	220	0.07
55–64	119,663	114	0.10
65–74	90,700	95	0.10
75+	41,907	71	0.17
Not recorded	331	0	0.00
Gender			
Males	135,478	178	0.13
Females	436,606	322	0.07
Not recorded	149	0	0.00
Ethnicity			
White	451,869	405	0.09
Asian or Asian British	20,106	25	0.12
Black or Black British	8,128	7	0.09
Other ethnic groups	5,657	9	0.16
Mixed	2,315	3	0.13
Chinese	1,059	0	0.00
Unknown	83,099	51	0.06
Deprivation (quintile)			
1-Most deprived	122,185	100	0.08
2	118,715	114	0.10
3	116,686	101	0.09
4	110,811	96	0.09
5-Least deprived	100,190	83	0.08
Not recorded	3,646	6	0.16
Tertiary centre			
No	461,346	386	0.08
Yes	110,887	114	0.10
Admission method			
Elective	510,260	435	0.09
Emergency	61,406	65	0.11
Transfer	431	0	0.00
Other	136	0	0.00
Acute cholecystitis (index admission)			
No	551,812	478	0.09
Yes	20,421	22	0.11
Acute pancreatitis (index admission)			
No	564,077	493	0.09
Yes	8,156	7	0.09
Year of index admission			
2001/2002	32,086	28	0.09
2002/2003	37,290	36	0.10
2003/2004	40,824	53	0.13
2004/2005	39,533	33	0.08
2005/2006	42,573	35	0.08
2006/2007	45,049	50	0.11
2007/2008	50,702	43	0.08
2008/2009	50,689	49	0.10
2009/2010	53,748	32	0.06
2010/2011	56,254	49	0.09
2011/2012	61,465	52	0.08
2012/2013	62,020	40	0.06
Converted			
Yes	25,513	254	1.00
No	546,720	246	0.04
No. procedures per institution (previous year, exc. 2001/2002)			
Low volume <200	113,391	82	0.07
Middle volume 200–500	286,943	258	0.09
High volume >500	139,813	132	0.09
No. procedures per consultant (previous year, exc. 2001/2002)			
Low volume <20	144,713	149	0.10
Middle volume 20–80	254,224	238	0.09
High volume >80	141,210	85	0.06

**Fig. 3:**
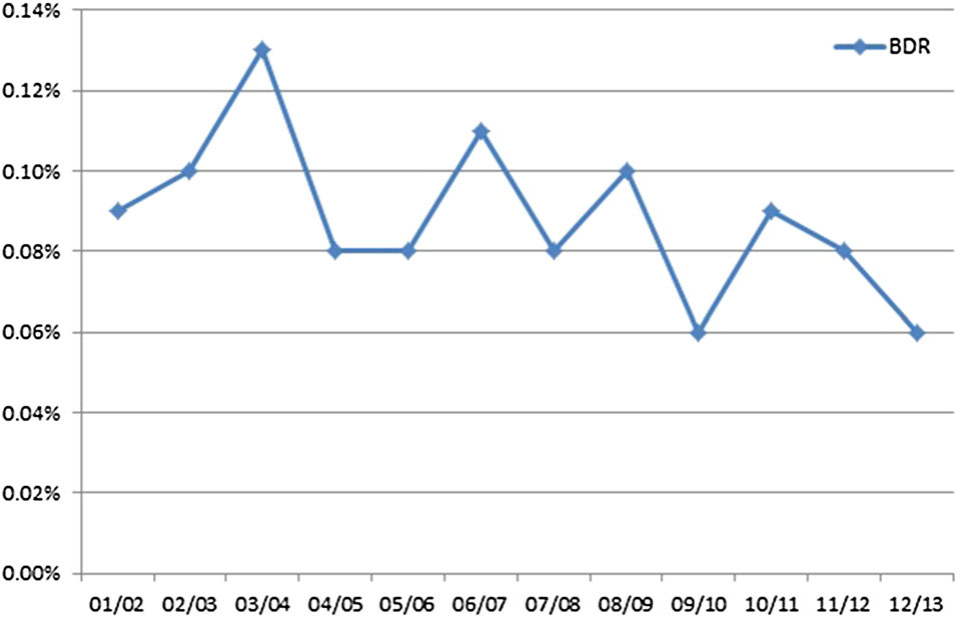
Bile Duct Reconstruction rate by study year

Five hundred patients underwent BDR within one year of a LC (0.09 %) (Table 3). Patients who underwent BDR following LC were 10 times more likely to die within a year of the index cholecystectomy (6 vs 0.6 %). There is a trend towards a lower rate of BDR (Fig. 3).

### Patient-related factors

Univariate analysis showed that patient-related factors including increasing age and male sex were significantly associated with bile duct reconstruction. However, multivariate analysis did not confirm these associations, suggesting that other factors may be responsible for these findings (Table 4). Only patients with acute cholecystitis who undergo LC on the index admission were found by both univariate and multivariate analyses to have an increased risk of BDR.

**Table 4:** Multivariate analysis of factors that may be associated with bile duct reconstruction following LC or attempted LC

Odds ratio—bile duct reconstruction		SE	*z*	*p* value	95 % CI	
Age group <55	1.00					
55–64	0.97	0.12	−0.27	0.787	0.76	1.23
65–74	0.86	0.12	−1.09	0.274	0.67	1.12
75+	1.22	0.18	1.33	0.185	0.91	1.63
Gender females	0.89	0.09	−1.1	0.269	0.73	1.09
Deprivation						
1-Most deprived	1.00					
2	1.24	0.18	1.52	0.128	0.94	1.64
3	1.13	0.17	0.85	0.396	0.85	1.51
4	1.15	0.17	0.96	0.335	0.86	1.55
5-Least deprived	1.11	0.17	0.65	0.513	0.81	1.51
No acute cholecystitis	0.48	0.11	−3.12	0.002	0.30	0.76
Acute pancreatitis	0.81	0.34	−0.5	0.620	0.36	1.83
Charlson score	0.94	0.08	−0.76	0.445	0.80	1.10
Cholangiography (index admission)	2.73	0.37	7.45	0.000	2.10	3.56
Converted procedure	22.89	2.33	30.75	0.000	18.75	27.94
No. procedures per consultant (prev. year, exc. 2001/2002)						
Low volume <20	1.00					
Middle volume 20–80	0.80	0.11	−1.64	0.100	0.62	1.04
High volume >80	0.56	0.10	−3.19	0.001	0.39	0.80
No. procedures per provider (previous year, exc. 2001/2002)						
Low volume <200	1.00					
Middle volume 200–500	1.07	0.18	0.37	0.710	0.76	1.49
High volume >500	1.31	0.24	1.44	0.150	0.91	1.89
Tertiary hospital	1.19	0.14	1.51	0.130	0.95	1.49
Consultant conversion rate—quartiles (previous year, exc. 2001/2002)						
1-Lowest quartile	1.00					
2	1.05	0.20	0.24	0.808	0.72	1.54
3	1.07	0.16	0.47	0.636	0.80	1.43
4-Highest	0.95	0.13	−0.36	0.721	0.74	1.24
Consultant cholangiography rate—tertiles (previous year, exc. 2001/2002)						
1-Lowest tertile	1.00					
2	1.17	0.18	1	0.318	0.86	1.58
3	0.69	0.09	−2.98	0.003	0.54	0.88

### Non-patient-related factors

Univariate and multivariate analyses showed that high-volume consultant caseload >80 LC/year is associated with a lower rate of BDR.

There was a strong association between conversion and BDR (*p* < 0.001), which may be due to surgeons converting when they suspect a BDI. Therefore, we used consultant conversion rate in the year before rather than conversion in an individual case. There was no association between consultant conversion rate in the previous year and BDR following LC or attempted LC.

Similarly, there was a strong association between the use of OTC and BDR in individual cases (*p* < 0.001). This may be due to surgeons using OTC when they suspect a BDI, but when consultants are divided into tertiles on the basis of their use of OTC in the year before the index case, those who use it more frequently have a lower rate of patients subsequently undergoing bile duct reconstruction, odds ratio 0.69 with 95 % CI (0.54–0.88).

Trust caseload volume was divided into low-volume providers <200 LC/year, intermediate-volume provider between 200 and 500 LC/year and high-volume providers, which perform more than 500 LC/year. Univariate and multivariate analyses did not show any association between Trust caseload volume and BDR.

There was no difference in the rate of BDR following LC or attempted LC if the index procedure was undertaken in an HPB centre as compared to a non-HPB centre.

A funnel plot was used to examine the rate of BDR following LC/attempted LC in individual trusts. All hospitals were within the 95 % confidence interval. Most BDRs were performed in the hospital in which an injury occurs rather than a regional centre (Table 5).

**Table 5:** Bile duct reconstruction at index or another hospital

Financial year (index admission)	No. bile duct reconstructions	Number performed at different hospital	% Bile duct repairs not in same hospital
2001/2002	28	10	35.7
2002/2003	36	12	33.3
2003/2004	53	15	28.3
2004/2005	33	14	42.4
2005/2006	35	16	45.7
2006/2007	50	24	48.0
2007/2008	43	13	30.2
2008/2009	49	19	38.8
2009/2010	32	11	34.4
2010/2011	49	18	36.7
2011/2012	52	21	40.4
2012/2013	40	22	55.0

## Discussion

This is the largest study of its kind in the literature that examines BDR following LC or attempted LC. It investigates all patients who underwent LC in England over a 12-year period. The apparent rate of BDR and therefore presumed bile duct injury is in keeping with published series (the previous literature for major injuries). Patient-related factors associated with BDR include cholecystitis on the index admission. Non-patient-related factors associated with a lower reconstruction rate include a high consultant cholecystectomy caseload and regular use of OTC. There is a tenfold increase in mortality at 1 year in patients who have undergone a BDR (at 1 year), demonstrating how serious this complication can be.

This study suggests that the incidence of BDR following LC in England is low (0.09 %) with only 500 cases over a 12-year period. Data from other registries show that the incidence of BDI in Germany is 0.1 % (172,368 LC) [2]; in Denmark 0.15 % (23,672 LC) [3]; in the USA between 0.06 and 0.5 % [4, 16]; in Finland 0.82 % (6733 LC) [5]; and in Sweden 1.5 % (51,041 LC) [6], although major BDI in this study accounts for only 0.1 %. However, researchers have to understand that different definitions of what constitutes BDI make comparative analysis difficult. Other reports from large single-centre studies (over 10,000 LC) showed the incidence of BDI is between 0.19 % [8] and 0.24 % [7]. There was a trend towards a reducing need for BDR during the study period, which may represent an increased awareness of methods of safe cholecystectomy.

The study has a number of limitations. There are no codes for BDI, and we therefore used codes for bile duct reconstruction. Other studies using registry data have used similar methodology [2–4, 17]. Patients who sustain a BDI and die without surgical intervention will not be included in this analysis. This study only identifies major duct injuries that require reconstruction, whereas minor injuries that require simple repair, drainage or ERCP and stenting are not included. Therefore, the study underestimates the incidence of BDI. Nevertheless, most minor injuries are associated with a lower rate of complications and lower long-term morbidity. However, the study does include those patients who fail to respond to ERCP and stenting or who develop stenosis of bile duct that requires delayed (within a year) surgical reconstruction.

The study uses HES data which are administrative data that rely on the accuracy of clinical coding. A recent systematic review shows that coding accuracy is improving and following the introduction of payment by results in 2002 the accuracy of coding for primary diagnoses has improved from 73.8 % (IQR 59.3–92.1 %) to 96.0 % (IQR 89.3–96.3) [18]. Further studies based on HES are cohort studies; they differ from the usual cohort studies in that they represent almost all activity within the area of study in England. One also has to consider the context of conclusions that are drawn from studies using HES. If findings are of a general nature, then even a relatively high coding error rate at some hospitals or even all hospitals will not detract markedly from the overall conclusions if significant deviation can be shown [19]. Thus, studies based on HES data may actually be good at dealing with research questions such as those posed in this study but are less good at identifying variations in care between individual trusts or clinicians [20]. We have not attempted to analyse the incidence of minor bile duct injuries as the coding issues are complex and we feel that it would be difficult to draw valid conclusions from the data.

Cholecystectomy is considered by many surgeons to be more difficult in male as compared to female patients, and this may lead to a higher complication rates. Our data showed male gender is associated with almost double the rate of BDR (0.13 %) compared to female patients (0.07 %). However, this difference becomes statistically insignificant when an adjustment is made for other factors.

Age has been shown to be an independent risk factor for BDI [21] and mortality following BDI [4]. Associated comorbidities, frailty, use of anticoagulants and previous abdominal surgery have been postulated to contribute to the increase in risk in the elderly [21]. Data from this study showed BDR following LC in elderly patients >75 years (0.17 %) was more than that in those below 55 years (0.07 %). However, multivariate analysis did not reveal any significant difference with age which implies that other factors are more important.

The calibre of the bile duct increases with age which may make simple repair easier in older patients [22, 23]. Whether simple suture repair of the bile duct can be accomplished depends on many other factors, for example, the presence of a clean laceration identified at the same time of surgery together with a wide calibre bile duct.

Several studies [24–26] have shown that bile duct injury repaired at an HPB centre is associated with a better outcome as compared to those repaired in a general hospital. Data from this study showed more than half of the injuries were repaired locally. Centralisation of HPB services has progressed rapidly in the UK with most major resections occurring in HPB centres during the study period. Many of those surgeons who used to perform resectional biliary surgery may still practice in their local hospital. Further some regions offer an outreach service where a BDI injury may be treated in the local hospital by a surgeon from the regional unit.

Most surgeons in the UK perform OTC selectively. Large studies based on registry data have produced conflicting results. While some show that the risk of BDI decreases when OTC is performed, [6, 17, 27–29] others, including a systematic review [30] show no benefit [31]. The study showed that surgeons who use OTC more frequently have a lower rate of BDR following LC.

The study did not show any difference in BDR following LC between low- and high-volume NHS providers or HPB centres and general hospitals, suggesting that all NHS providers deliver a satisfactory cholecystectomy service. However, it appears consultant caseload is an independent risk factor for BDR following LC. Surgeons who perform 80 LC/year or more have a lower rate of BDR than low-volume surgeons. Further BDR appears to be more common in patients who undergo cholecystectomy on an index emergency admission with acute cholecystitis.

These results suggest that more widespread use of OTC could also help to reduce the incidence of BDI. They do not support centralisation of cholecystectomy services; however, they do suggest that to avoid major bile duct injury the development of dedicated teams in each hospital with an adequate LC caseload (>80) may help to reduce the incidence of this complication and further suggests that the occasional operator should reconsider their practice especially in emergency patients.
